# M. Bourgery on the Minute Structure of the Lungs in Man and the Mammalia

**Published:** 1842-11-05

**Authors:** 


*On the minute Structure of the Lungs in Man and the Mammalia.* By M. Bourgery.—The peculiarities of M. Bourgery's views relate chiefly to the mode in which the minutest bronchial tubes terminate. "Each lobule," he says, "receives a single central bronchial branch, which forms the common tree of its air-tubes; or, if the lobule be large, two or even three of these branches may enter into it. The smallest of these lose themselves laterally in the manner presently to be described; a single tube, which is the continuation of the trunk, reaches the peripheral base of the lobule, and there ramifying, turns towards one of its angles, which forms the terminal summit. Proceeding from this decreasing central tree there arise from every side, in alternate succession and in star-like radiation, secondary branches, which I have named *bronchial ramified canals*, the ultimate expansion of the tracheal tree, beyond which the *labyrinthic* arrangement commences."

This labyrinthic arrangement is made up of numberless minute canals, (*ærial labyrinthic canals*,) arising from the bronchial ramified canals, running tortuously in every direction, and branching and anastomosing in the most multiform manner. They thus inclose numberless irregular spaces, in which, as well as on their walls, the blood-vessels ramify and form their capillary net-work. They give the idea of a space all divided with thousands of tortuous branchings, everywhere continuous with itself, and where there is no termination except the one common orifice of entry and exit in the trunk of the bronchial tree of each lobule. In no observed instance was there anything like a cul-de-sac or cæcal termination of any of the canals; "whatever point or surface he observed, there are flexuous canals anastomosing in every plane, but nowhere are there any straight canals without anastomoses or any vesicles." The sinuous canals, moreover, are from space to space constricted, not, as Willis supposed, by ligamentous fibres, but by annular vessels, circumscribing in their intervals *loculi*, at the bases of which are the orifices of other labyrinthic canals, and giving that appearance of chains of cellules which forms the basis of the theories of Malpighi and Helvetius. In such canals as these, the ramified bronchial canals, after giving off great numbers of them from their sides, ultimately, by dividing twice or three times, terminate; and their terminal branches enter into the common labyrinth, comporting themselves like the tubes given off from their sides.—*British and Foreign Med. Review*. October, 1842. *From Gazette Médicale*. Juillet 16, 1842.

